# The Cellular Localization of the p42 and p46 Oligoadenylate Synthetase 1 Isoforms and Their Impact on Mitochondrial Respiration

**DOI:** 10.3390/v11121122

**Published:** 2019-12-04

**Authors:** Stig Skrivergaard, Monica Skou Jensen, Tine Breckling Rolander, Tram Bao Ngoc Nguyen, Amanda Bundgaard, Lene N. Nejsum, Pia M. Martensen

**Affiliations:** 1 Department Molecular Biology and Genetics, Aarhus University, 8000 Aarhus C, Denmark; skrivergaardstig@gmail.com (S.S.); monicaskou@gmail.com (M.S.J.); tine_bc@hotmail.com (T.B.R.); tramnguyeen@hotmail.com (T.B.N.N.); 2 Department Bioscience, Aarhus University, 8000 Aarhus C, Denmark; ammagabu@bios.au.dk; 3 Department Clinical Medicine, Aarhus University, 8200 Aarhus N, Denmark; nejsum@clin.au.dk

**Keywords:** OAS1, high resolution respirometry, oxygen consumption rate, mitochondrial transmembrane potential, oroboros, CaaX motif, cellular localization, type 1 inteferon, ISG

## Abstract

The importance of the IFN-induced oligoadenylate synthetase (OAS) proteins and the OAS/RNase L pathway in the innate response against viral pathogens is well-established, however the observed differences in anti-viral activity between the human OAS1 p46 and p42 isoforms are not fully understood. The protein expression of these isoforms is determined by the SNP rs10774671, either being an A or a G allele resulting in expression of either the p42 or the p46 isoform. Using fluorescence microscopy and immunoblot analysis of fractionated cell samples, we show here that the CaaX motif is of key importance to the cellular localization. The OAS1 p42 isoform is mainly located in the cytosol, whereas the p46 isoform with a C-terminal CaaX motif is translocated to membranous organelles, like the mitochondria. We furthermore observed differences between p42 and p46 in their effect on mitochondrial physiology using high resolution respirometry and fluorometry. Overexpression of OAS1 p42 and IFN-β treatment of HeLa cells (AA genotype) resulted in significantly increased respiration, which was not seen with p46 overexpression. The difference in subcellular localization and mitochondrial effect of these two OAS1 isoforms might help to explain the anti-viral mechanisms that differentiate these proteins.

## 1. Introduction

The oligoadenylate synthetase (OAS) family genes encode four different types of proteins: OAS1, OAS2, OAS3, and OAS-Like (OAS-L) of which the OAS1, OAS2, and OAS3 are enzymatically active while the OAS-L is not [1]. The structure and mechanistic function of the OAS1 protein is well-described [2,3], as well as the canonical downstream anti-viral properties of the OAS-activated RNase L pathway [4,5,6]. The OAS proteins are some of the key proteins upregulated during type I and type III IFN response following viral infection [7].

The human OAS1 gene is 25,414 base pairs long and consists of eight exons. Of these exons, the first five exons from the 5′ end are translated into the core protein while the latter three exons enable alternative splicing into the six known protein isoforms of OAS1. Two of these isoforms are the p42 and p46 variants which are the focus of this study. The two isoforms are so named due to their theoretical size of ~42 and ~46 kDa and the expression pattern between these two variants is related to one single nucleotide polymorphism (SNP), the SNP rs10774671 [8,9]. The SNP is an A/G variation located in the splice acceptor site of exon 7 which results in either the A allele expressing the p42 isoform or the G allele expressing the p46 isoform. 

The rs10774671 SNP and in extension the discrepancy between the p42 and p46 isoforms have been directly associated with several viral diseases including West Nile Virus infections [10], susceptibility to hepatitis B virus (HBV) infections and Sjögren’s syndrome development [11], liver fibrosis progression and response to interferon therapy during hepatitis C virus (HCV) infections [12,13] as well as in tuberculosis (TB) [14]. In all of these instances, it is the G allele (i.e., the OAS1 p46 isoform) that confers resistance, while the p42 variant is associated with a higher risk in these infectious diseases. It has also been shown that the p46 isoform, but not the p42 isoform, facilitate RNase L-dependent anti-viral activity against HCV in cultured Huh7 cells [15]. 

The subcellular localization of the two OAS1 isoforms also seems to be different. We have previously observed that the OAS1 p46 isoform is localized in the mitochondria, whereas the p42 is more evenly distributed in the cytosol [9]. We have hypothesized that this difference could be due to the presence of a CaaX motif (C = Cysteine, a = aliphatic amino acid, X = terminal amino acid) in the C-terminal of only the p46 isoform, which could be important in directing the protein to the mitochondria. Generally, a CaaX motif serves as a peptide signal for a posttranslational modification termed prenylation, which is the covalent binding of an isoprenoid lipid on the cysteine residue. This terminally located lipid-modified cysteine can facilitate intracellular trafficking and translocation to a membrane [16,17]. The RNase L enzyme has also been found in the mitochondria where it can degrade mitochondrial RNA and facilitate IFN-α-induced apoptosis [18], thereby making a complete mitochondrial OAS1/RNase L-system plausible.

The mitochondria are the powerhouses of the cell, being the organelles where the major part of ATP synthesis takes place. The enormous demand for ATP is supplied by a complicated albeit dynamic interplay between protein pumps and electron carriers collectively called the electron transport system (ETS) resulting in oxidative phosphorylation (OXPHOS) and the synthesis of ATP [19]. The proton motive force driving the OXPHOS consists of two parameters, the chemical pH gradient (ΔpH) and the transmembrane potential (Δψ) (i.e., a charge gradient). As the Δψ significantly exceeds the ΔpH in mitochondria [20], the mitochondrial transmembrane potential (Δψm) will be used later as the measure of proton motive force and as an indicator of the general constitution of the mitochondria and the ETS-supported OXPHOS. 

Mitochondria are not only paramount in cell life, they are also positioned at the heart of cell death [21]. Apoptosis, the unique process of programmed cell death, is an important cellular event in multicellular organisms. The mitochondria have several crucial roles in this mechanism mainly involving the permeabilization of the outer membrane, resulting in cytochrome c release, which are stimulated by death signals, e.g., DNA damage and endoplasmic reticulum (ER) stress. [22]. The mitochondria are also important in anti-viral immune responses. For example, the mitochondrial anti-viral signaling (MAVS) protein can regulate the powerful NF-κB and interferon pathways after interacting with the viral RNA-sensing protein retinoic acid-inducible gene I (RIG-I), which are activated by virus-derived dsRNA [23]. Because of the key importance of mitochondria in apoptosis, cell metabolism and immune response, they are also a highly attractive target for many viral pathogens to manipulate in various manners to increase overall pathogen survival and thus pathogenesis [24]. 

If the OAS1 p46 isoform is associated with the mitochondria and not the p42 variant, it might give insight into an unknown anti-viral mechanism and explain the observed differences between p46 and p42 in protection against viral pathogens.

The aim of this study was to determine the spatial distribution of the OAS1 p42 and the p46 isoforms within the cell and to establish any correlation between the CaaX motif and the OAS1 localization. The localization study has been performed using immunofluorescence cytochemistry analyses supported by digitonin-mediated fractionation combined with immunoblot analysis. The physiological effects of the OAS1 isoforms as well as of interferon β on the mitochondria was analyzed using a simultaneous multi step measurement of respiration and mitochondrial transmembrane potential. This has been achieved by a specifically designed protocol using permeabilized cells in different inducible respiration states via the Oroboros Oxygraph-2k high resolution respirometry and fluorometry capabilities.

Our work highlights the significant differences between the OAS1 p46 and p42 isoforms in the context of subcellular localization and their effect on mitochondrial physiology. The physiological impact of IFN-β and p42/p46 overexpression on cellular respiration was determined in HeLa and HT1080 cells. We have furthermore established that this difference in localization is a direct consequence of the C-terminal CaaX motif. Overall, these results improve upon the knowledge of the OAS1 system and could further add to the understanding of the differences in antiviral activity between the OAS1 p42 and p46 isoforms previously observed. 

## 2. Materials and Methods

### 2.1. Cell Lines

HeLa (ATTC: CCL-2) and HT1080 (ATCC: CCL-121) cells were cultured as adherent cells at 37 °C with 5% CO_2_ in Dulbecco’s modified Eagle’s medium with high glucose and with sodium pyruvate (Life Technologies Europe BV, Nærum, Denmark) with added 10% sterile-filtered fetal bovine serum (FBS) (Sigma-Aldrich, Søborg, Denmark ) and 1% Penicillin-Streptomycin (Pen-Strep) solution (Sigma-Aldrich) at 37 °C and 5% CO2. The human HT1080 and HeLa cell lines used have the AG genotype and the AA genotype, respectively, regarding the SNP rs10774671 [9]. For interferon treatments, both HeLa and HT1080 cells were grown to ~80% confluence and were treated with 1000 U/mL IFN-β or left untreated for 24–48 h before analysis. Both cell-lines were tested and found mycoplasma free. 

### 2.2. High Resolution Respirometry (HRR) 

The high resolution respirometry (HRR) protocol used was adapted from [25]. First, calibration of the Oroboros Oxygraph-2k (Oroboros Instruments, Innsbruck, Austria) was performed for approximately 30 minutes allowing cell culture medium to achieve oxygen saturation and 37 °C. Calibrated oxygen levels were determined by the DatLab7 Oroboros software and a solubility factor (FM) of 0.89 was used for this medium. The cells were counted and the cell suspension was diluted with culture medium to obtain a final concentration of 1 × 10^6^ cells/mL. The incubation media used for calibration was then removed and 2 mL of the cell suspension were added to the chambers. Measurements were performed at 37 °C with sample mixing at 750 rpm by PVDF-coated magnets. After approximately 10 min the ROUTINE respiration was stabilized. The standard substrate-uncoupler–inhibitor–titration (SUIT) protocol was then initiated and 1 μL of 5 mM oligomycin (Sigma-Aldrich) was injected into the chambers to a final concentration of 2.5 μM to induce the LEAK respiration (non-phosphorylating resting state). After stabilization, the titration with Carbonyl Cyanide m-ChloroPhenylhydrazone (CCCP, Sigma-Aldrich) or Carbonyl Cyanide 4-(triFluoro-methoxy)Phenylhydrazone (FCCP, Sigma-Aldrich) in 0.5 μM steps (1 μL 1 mM CCCP) was performed until the highest oxygen consumption rate (OCR) was achieved. Then 1 μL of 5 mM antimycin A (Sigma-Aldrich) was injected to a concentration of 2.5 μM to induce the ROX state (residual oxygen consumption). The oxygen consumption rate (OCR) values was marked in DatLab7 to give the median of the marked area.

### 2.3. Simultaneous Measurement of HRR and Fluorometry

The Oroboros was calibrated as described above with a respiration buffer containing 80 mM KCl, 10 mM Tris-HCl, 3 mM MgCl_2_, 1 mM EGTA, and 5 mM KH_2_PO_4_. The solubility factor FM was set to 0.96. After oxygen sensor calibration, the fluorescence sensors with safranin filters were attached and safranin was titrated up to a final concentration of 2.5 μM. Linearity of safranin signal was established, and the raw fluorescence signal was calibrated. Cells were trypsinized and diluted in cell culture media without phenol red. The cell suspension was centrifuged for 10 min at 200× *g*, the supernatant was discarded, and the cells were resuspended in 1 mL of ice-cold respiration buffer. The cell suspension was transferred to a 1.5 mL microcentrifuge tube and was centrifuged at 200× *g* for 10 min at 4 °C. The supernatant was removed carefully and 100 μL of ice-cold respiration buffer was added. The cells were counted, and the required volume of cell suspension was added to the chambers to obtain a final concentration of 1 × 10^6^ cells/mL. Cells should not be in suspension for more than one hour prior to measuring. The cells were permeabilized using 40 μM of digitonin (Sigma-Aldrich). The extended SUIT protocol could then be initiated by injecting the next substrate or inhibitor when both OCR and fluorescence values were stabilized. The SUIT chemicals were injected in the following order to obtain final concentrations of: 2 mM malate + 10 mM glutamate (Sigma-Aldrich), 1 μM rotenone (Sigma-Aldrich), 10 mM succinate (Merck Millipore, Darmstadt, Germany), 4 mM ADP (Sigma-Aldrich), 2.5 μM oligomycin, 25 nM CCCP titration steps, and 2.5 μM antimycin A. Data collection was performed with DatLab7.

### 2.4. Transfection of Cells

Cells were seeded in 6-well plates (for cell fractionation), on coverslips (for immunocytochemistry) or in T75 flasks (for HRR). Linear polyethyleneimine (PEI) Mw 25.000 (Polysciences, Inc., Hirschberg an der Bergstrasse, Germany) were mixed with Opti-MEM (Gibco®, ThermoFisher Scientific, Roskilde, Denmark) and incubated for 5 min before mixing it with the plasmid of interest also in Opti-MEM. The optimal ratios of DNA:PEI were found to be 1:6 (*w*/*w*) for both HeLa and HT1080 cells. DNA:PEI complexes were allowed to form for at least 15 minutes at room temperature, before it carefully was added to the cells with a confluence of ~80% in culture media without Pen-Strep. Cells were incubated for 5 h and the media was changed. The cells were then harvested and further analyzed after 24 h (for cell fractionation and immunoblotting), 28 hours (for immunocytochemistry), or 48 h (for HRR).

### 2.5. Plasmids

The CaaX (CTIL) motif was deleted in the p46 encoding plasmid and added to the p42 encoding plasmid by Quick Change Site-Directed Mutagenesis (Stratagene, La Jolla, California, USA). Primers were designed to anneal to either side of the region to be deleted or added.

Forward primer (p46ΔCaaX): 5′ - CAG AAG AGG ACT GGA CCT GAA TGC CAG TGC ATC - 3′

Reverse primer (p46ΔCaaX): 5′ - GAT GCA CTG GCA TTC AGG TCC AGT CCT CTT CTG - 3′

Forward primer (p42CaaX): 5′-AA GCT TGC ACC ATC CTC TGA GAC ATA TAG CTG GAG ACC ATT CTT-3′

Reverse primer (p42CaaX): 5′-GAG GAT GGT GCA AGC TTC ATG GAG AGG GGC AGG GAT GAA TG-3′

PCR amplifications were performed using the above primers. The reactions were in a total volume of 30 uL in 1 × Pfu reaction buffer with 3 mM MgSO_4_, 250 μM dNTP mix, 1 μL Pfu DNA polymerase (Invitrogen, ThermoFisher Scientific, Roskilde, Denmark), 125 ng of each primer and 40 ng of template DNA. After an initial denaturation at 95 °C for 30 s, the thermal cycle was programmed for 30 s at 95 °C for denaturation, 60 s at 65 °C for annealing and 12 min at 72 °C for elongation. This cycle was repeated 18 times. One microliter of Dpn1 restriction endonuclease was added to the PCR products and incubated at 37 °C for 2 h. Positive colonies were selected on 100 μg/mL ampicillin agar plates. 

For the EGFPp42 and EGFPp46 fusion plasmids, PCR products were amplified from the p42wt or the p46 wt encoding plasmids using the following primers:

Forward primer (for both constructs): 5′-TTAAGTCTCGAGGAAACTTGGGTGGTGGA-3′

Reverse primer (pEGFPp42): 5′-AGCTTACTGCAGTCAAGCTTCATGGAGAGG-3′

Reverse primer (pEGFPp46): 5′- GACTAACTGCAGTCAGAGGATGGTGCAG-3′

The PCR products were cloned into the pEGFP-C2 vector (ClonTech, Takara Bio Europe SAS, Saint-Germain-en-Laye, France) using Xho1 and Pst1 restriction sites, and colonies were selected on 50 μg/mL kanamycin agar plates. 

**Figure 1:**
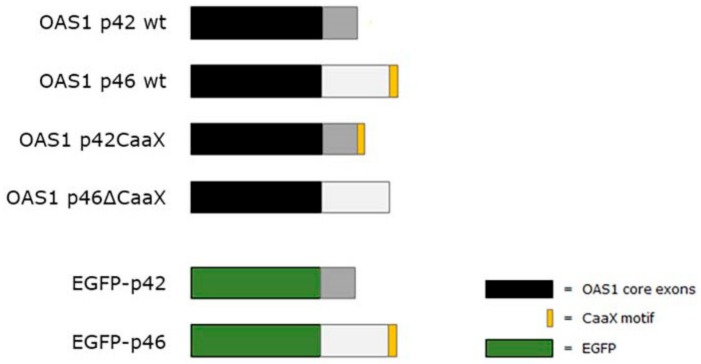
Oligoadenylate synthetase (OAS)1 and EGFP constructs used for transient transfections. The diagram shows the different types of OAS1 and EGFP constructs used to induce overexpression of the translated proteins for the high resolution respirometry (HRR) and localization experiments. All the OAS1 types consist of 5 core exons (black). The wild type p42 (grey) and p46 (white) isoforms are different in their C-terminal ends and are modified to include or exclude the CaaX motif (yellow). Full-length EGFP (green) is fused to either the C-terminal of p42 or p46 as indicated.

All constructs were verified by sequencing and are illustrated in Figure 1.

### 2.6. Immunofluorescence Microscopy

HeLa cells were seeded on poly-D-Lysine coated coverslips and transfected with plasmids encoding either p42, p42CaaX, p46, p46ΔCaaX, EGFPp42, EGFP46, or control plasmids using the PEI method (see above). After 28 h, 50 nM of Mitotracker Red CMXRos (Molecular Probes, Invitrogen, ThermoFisher Scientific, Roskilde, Denmark) was added to the cells for 20 min. Cells were then fixed with 4% paraformaldehyde, washed twice in PBS, permeabilized with 0.05% Triton X-100 for 10 min, washed twice with PBS and finally blocked with 3% BSA in PBS-T. The coverslips were then either incubated with a specific human OAS1 (13-4-10) antibody (1:500, a kind gift from Dr. Shawn P. Iadonato) followed by incubation with a FITC-conjugated anti-mouse antibody (1:250, Sigma-Aldrich) for the OAS1 expressing plasmids or no antibodies (for the EGFP constructs). Nuclei were stained with 20 μg/mL Hoechst 33342 (Invitrogen). The coverslips were mounted with Glycergel Mounting Medium (DAKO) containing 2.5% (*w*/*v*) antifade Dabco 33-LV (Sigma-Aldrich). Regular wide field epifluorescence microscopy was performed using a Nikon Ti Eclipse inverted microscope equipped with a motorized stage, a 100× objective, and the TI-ND6-PFS-S Perfect Focus System controlled by NIS-Elements software (Nikon, Ramcon A/S, Birkerød, Denmark). 12 bitimages were captured with a Zyla 5.5 sCMOS camera (Andor Technology Ltd., Belfast, UK). The epifluorescence light source was a pE-300white LED unit (CoolLED, Ramcon A/S, Birkerød, Denmark) and appropriate filter sets were used. The images were analyzed with ImageJ Fiji [26]. Brightness-contrast was adjusted differentially for for each image except for Supplementary Figure S4, in which the images were adjusted identically by setting the minimum displayer value to 800.

### 2.7. Cell Fractionation

Fractionation was performed as previously described [27]. Briefly, cells were harvested and centrifuged at 200× *g* for 10 minutes at 4 °C in microcentrifuge tubes, to pellet the cells. The cell pellet was washed in ice cold PBS and pelleted again. The supernatant was discarded and the cells were resuspended in 100 μL of ice-cold respiration buffer with 40 μM digitonin and 1 μL/mL protease inhibitor (P3340, Sigma-Aldrich). Cells were then incubated on ice for 15 minutes before centrifugation at 2000× *g* for 10 min at 4 °C. The resulting supernatant was the cytosolic fraction (C) diffused from the permeabilized plasma membrane. The supernatant was collected without disturbing the pellet. The cell pellet was then resuspended in 100 μL IgePal lysis buffer containing 50 mM Tris-HCl (pH 8.5), 150 mM NaCl and 1% IgePal (CA-630) with protease inhibitor (P3340, Sigma-Aldrich) and was incubated for 30 min on ice. The samples were centrifuged at 7000× *g* for 10 min at 4 °C and the supernatant was collected. This supernatant was the mitochondrial fraction (P) (also containing proteins from the ER, Golgi and other subcellular organelles). Fractionated samples were analyzed by immunoblot immediately after or were stored at −80 °C. 

### 2.8. Immunoblot Analysis

Protein samples were subjected to 10–14% SDS-PAGE and were transferred to PVDF membranes. Blocking of membranes was performed with 5% (*w*/*v*) skimmed milk powder (EASIS A/S, Åbyhøj, Denmark) in TBS with 0.05% (*v*/*v*) Tween 20 (TBS-T) (Merck Millipore). Proteins were detected using the following antibodies diluted in 5% (*w*/*v*) skimmed milk powder in TBS-T: A human OAS antibody recognizing all isoforms of human OAS proteins (1:2000) [28], a cytochrome C antibody (1:2000; BD Biosciences), a Calnexin antibody (1:5000; Sigma-Aldrich), an actin antibody (1:5000; Sigma-Aldrich) or an anti-EGFP antibody (a kind gift from Dr. Koh-ichi Nagata; 1:5000). Appropriate HRP-conjugated secondary antibodies were used, either goat-anti-rabbit (1:2000; Dako Denmark A/S, Glostrup, Denmark) or sheep-anti-mouse (1:5000; GE Healthcare, Brøndby, Denmark). Detection was performed using the ECL Prime detection reagent (GE Healthcare) and visualized by a CCD camera (Amersham Imager 600, GE Healthcare). Stripping of PVDF membranes were performed by letting the membranes dry out followed by a re-activation step with 96% ethanol for 5 min on a shaking table. Quantification of band intensities were performed using the ImageQuant software (GE Healthcare).

### 2.9. Statistical Analysis

All quantification data from immunoblotting, HRR and fluorometry were analyzed in GraphPad Prism 6/8. Statistical analysis between two samples with one grouping variable was performed using an unpaired t-test, while a multiple t-test using the Holm-Sidak method was performed between data sets with several grouping variables. The P-values obtained were two-tailed and the threshold of significance was defined as *P* < 0.05. All data are represented in column bar graphs with mean values and error bars calculated by the standard error of the mean (SEM).

## 3. Results

### 3.1. Effect of IFN-β on Mitochondrial OCR in HeLa and HT1080 Cells

**Figure 2:**
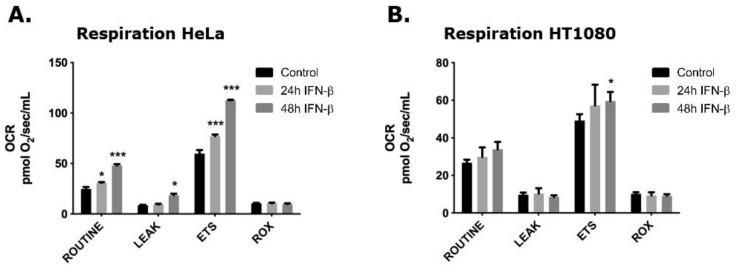
Respiration in Hela and HT1080 cells. Cells were treated with 1000 U/ml IFN-β for 24 or 48 hours, as indicated. Controls were untreated cells. Oxygen consumption rates of (**A**) Hela and (**B**) HT1080 cells were measured according to the SUIT protocol for High Resolution Respirometry after establishment of ROUTINE respiration in cell culture medium. Data are presented as mean values of independent experiments and error bars represent SEM. Number of replicates for Hela cells were: *n* = 6 for controls and IFNβ 24 h, and *n* = 4 for IFNβ 48 h. Number of replicates for HT1080 cells were: *n* = 12 for controls, *n* = 4 for IFNβ 24 h, and *n* = 6 for IFNβ 48 h. * indicates *P* < 0.05, ** indicates *P* < 0.001, and *** indicates *P* < 0.0001 compared with control cells.

We first analyzed the effect of IFN-β response on cellular respiration of the human HeLa and HT1080 cell lines using high resolution respirometry (HRR). Very little literature describes the effect of type I IFN on respiration in general and the direct effect on these cell lines have to our knowledge not been established. However, type I IFN is thought to promote glycolysis and decrease oxidative consumption [29]. Therefore, it was interesting to see that the oxygen consumption rate (OCR) in HeLa cells was increased by approximately two-fold after 48 hours of IFN-β treatment in three different respiration states, i.e., ROUTINE, LEAK, and ETS (Figure 2A). The ROUTINE state is the basal respiration in normal media, while the LEAK state is induced by the ATP-synthase inhibitor oligomycin signifying the non-phosphorylating resting state and the degree of proton leak across the inner mitochondrial membrane. ETS state is the maximal OCR obtained when membrane potential is dissipated by the uncoupler CCCP (Carbonyl Cyanide m-ChloroPhenylhydrazone). This results in a noncoupled mitochondrial state in which there are no limitations to proton availability thereby enabling the determination of the maximum capacity of the electron transport system (ETS). The residual oxygen consumption (ROX) state is induced by the Complex III inhibitor, antimycin A, and is based on oxygen consuming enzymes and ROS production. A representative of an HRR measurement of HeLa cells can be seen in Supplementary Figure S1. The OCR of HT1080 cells was also significantly increased after 48 hours of IFN-β treatment in the ETS respiration state (Figure 2B), albeit much less than in HeLa cells. The OCR values in the different respiration states of HeLa and HT1080 cells are very similar in the untreated controls, however when responding to IFN-β the two cell lines show significantly different behaviors. 

**Figure 3:**
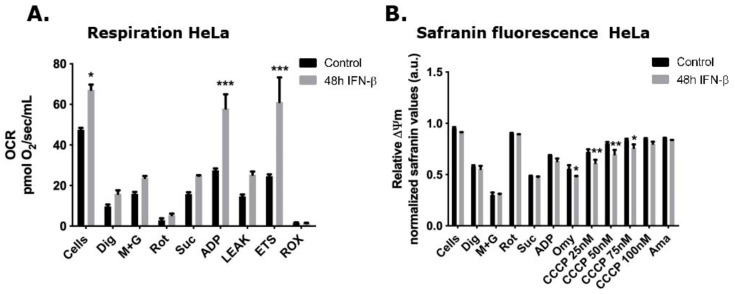
Respiration and mitochondrial membrane potential in IFN-β treated HeLa cells. Cells were treated with 1000 U/mL IFN-β for 48 h or left untreated. Simultaneous oxygen consumption rates (**A**) and levels of fluorescent safranin (**B**) were measured according to the extended SUIT protocol for High Resolution Respirometry. Data are presented as mean values of two independent experiments and error bars represent SEM. Safranin values are normalized against highest initial fluorescence before cell addition and are in arbitrary units (a.u.). Note that a lower safranin value equals a higher Δψm.* indicates *P* < 0.05, ** indicates *P* < 0.001, and *** indicates *P* < 0.0001 compared with control cells.

To further analyze the effect of IFN-β on mitochondrial physiology we designed an extended HRR protocol with a simultaneous fluorescence measurement capable of determining the mitochondrial transmembrane potential (Δψm). This protocol enabled additional respiration steps to be analyzed in a specifically designed respiration buffer by addition of specific substrates (malate + glutamate and succinate) due to the digitonin permeabilization. In this set-up we observed a significant increase in OCR in the basal and ETS respiration states and furthermore at the ADP step after 48 hours of IFN-β treatment in HeLa cells (Figure 3A). This ADP step signifies the OXPHOS capacity when ATP-synthesis is stimulated by ADP addition (sustained by succinate as an ETS-linked substrate). Using the safranin fluorescence, we also observed a significant increase in Δψm at the oligomycin step (LEAK) and in the three first titration steps of CCCP (25, 50, and 75 nM) for the induction of the ETS state (Figure 3B). In this experiment a lower safranin signal signifies a higher Δψm in the cells. The raw data of one of the experiments can be seen in Supplementary Figure S2.

**Figure 4:**
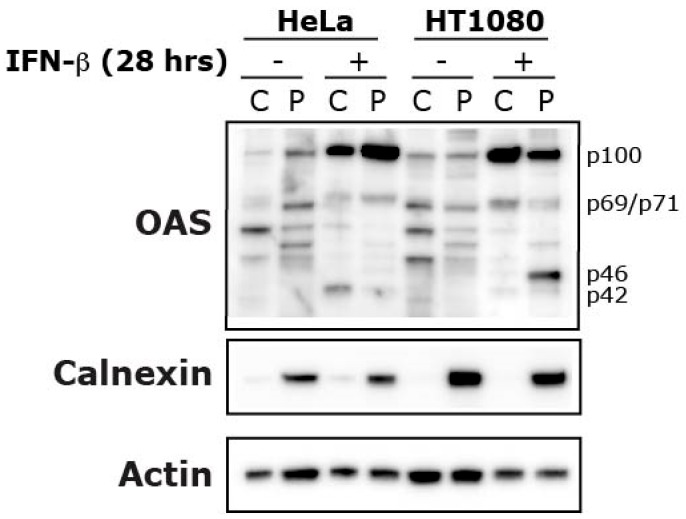
Cellular localization of OAS proteins in IFNβ treated HeLa and HT1080 cells. Cells were treated with 1000 U/mL IFN-β (+) for 28 h or left untreated (−) and harvested by centrifugation. The cells were lysed and fractionated into the cytosol (C) and the pellet (P). The P fraction contains proteins from mitochondria, endoplasmic reticulum, Golgi, and other internal organelles excluding the nucleus, which was separated in a centrifugation step. Protein extracts were subjected to immunoblot analysis using antibodies against the indicated proteins. The OAS antibody recognizes the different isoforms of OAS: OAS1 (p42 and p46), OAS2 (p69/p71), and OAS3 (p100).

To visualize the inherent differences in OAS protein expression between the HeLa and HT1080 cells, we analyzed protein extracts using immunoblotting analysis after IFN-β treatment (Figure 4). The protein samples from the cytosol (C) and pellet (P) (containing membranous organelles except the nucleus) were retrieved using digitonin permeabilization. The OAS isoforms OAS1 (p42 and p46), OAS2 (p69/p71) and OAS3 (p100) was detected using an anti-OAS antibody. There is a clear difference between the two cell lines after IFN-β treatment. HeLa cells only express the p42 isoform, while the HT1080 cells express both isoforms, albeit a higher amount of the p46 isoform compared with p42 (Figure 4). This is due to Hela cells being the AA genotype and HT1080 cells being the AG genotype regarding the rs10774671 SNP, the deciding factor of the pre-mRNA splicing and the OAS1 isoform ultimately being expressed. These differences can also be seen on the OAS1 mRNA levels in Supplementary Figure S3. Furthermore, it can be noticed that the spatial distribution of OAS1 p46 and p42 is different, as the p42 is mainly found in the cytosolic fraction, while the p46 band is more intense in the pellet fraction. This difference in localization will be analyzed in more detail later. Both cell lines express the OAS2 and OAS3 isoforms after IFN-β treatment, and these larger OAS isoforms seem to be distributed between the cytosol and membrane fractions, although the distribution is slightly different between the two cell lines.

### 3.2. Effect of OAS1 p46 and p42 on Mitochondrial OCR

**Figure 5:**
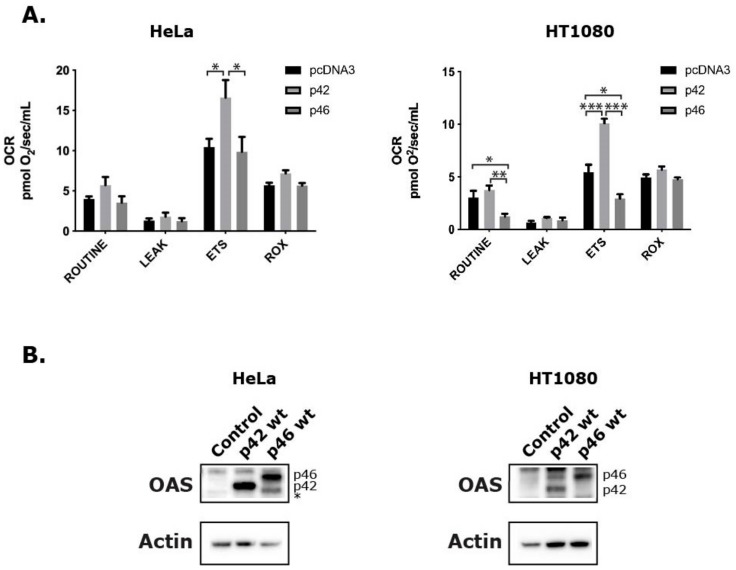
Respiration in HeLa and HT1080 cells upon overexpression of the OAS1 isoforms p42 and p46. Cells were transfected with plasmids encoding p42 and p46, or a control plasmid (pcDNA3) for 48 hours, as indicated. (**A**) Oxygen consumption rates were measured according to the SUIT protocol for High Resolution Respirometry after establishment of ROUTINE respiration in respiration buffer. Transfection efficiencies were around 30% for HeLa cells and 20% for HT1080 cells. Cell concentrations were 0.5 × 10^6^ cells/mL for HeLa cells and 0.3 × 10^6^ cells/mL for HT1080 cells. Data are presented as mean values of independent experiments (*n* = 8 for HeLa cells and n ≥ 3 for HT1080 cells) and error bars represent SEM. * indicates *P* < 0.05, ** indicates *P* < 0.001, and *** indicates *P* < 0.0001. (**B**) Protein extracts were analyzed by immunoblotting using antibodies against the indicated proteins. The anti-OAS antibody recognizes all the OAS isoforms. The positions of the p42 and p46 isoforms are indicated. * Indicates the position of a cleaved form of p46.

The difference in respiration between the HeLa and HT1080 cells after IFN-β treatment that we observed in Figure 2 was quite significant. This might be due to their OAS1 rs10774671 SNP genotype and the OAS1 p46 and p42 isoforms produced (Figure 4), although other explanations could be put forward. To test how the expression of p42 or p46 influence the mitochondrial respiration, we transiently transfected both cell types with plasmids encoding either the OAS1 p46 or the OAS1 p42 isoform, or with an empty vector as control. After transfection we performed HRR measurements and observed distinct differences (Figure 5A). In HeLa cells we observed that the p42 isoform conferred a significant increase in OCR in the ETS state when compared to the control and p46 expressing cells. In the HT1080 cells we observed a very interesting pattern in which the p46 significantly decreased the OCR, while the p42 significantly increased the OCR when compared to the control in the ETS state. The p46 isoform also decreased the OCR significantly in the ROUTINE state. This decrease, only observed in the HT1080 cells, could indicate that the p46 response is cell-type specific. In Figure 5B, an immunoblot can be seen showing the levels of p42 and p46 in transfected cells, as well as the basal level of OAS1 expression after transfection with the empty pcDNA3 vector. Only in the HT1080 cells, a small amount of p46 can be seen after transfection with the empty vector, and also expression of p46 in the cells transfected with the p42 expressing plasmid. The opposite effect of the OAS1 p46 and p42 on the mitochondrial physiology is indeed interesting and could have important implications in mitochondrial health and anti-viral protection. 

### 3.3. Subcellular Localization of OAS1 p46 and p42 and the Importance of the CaaX Motif

**Figure 6:**
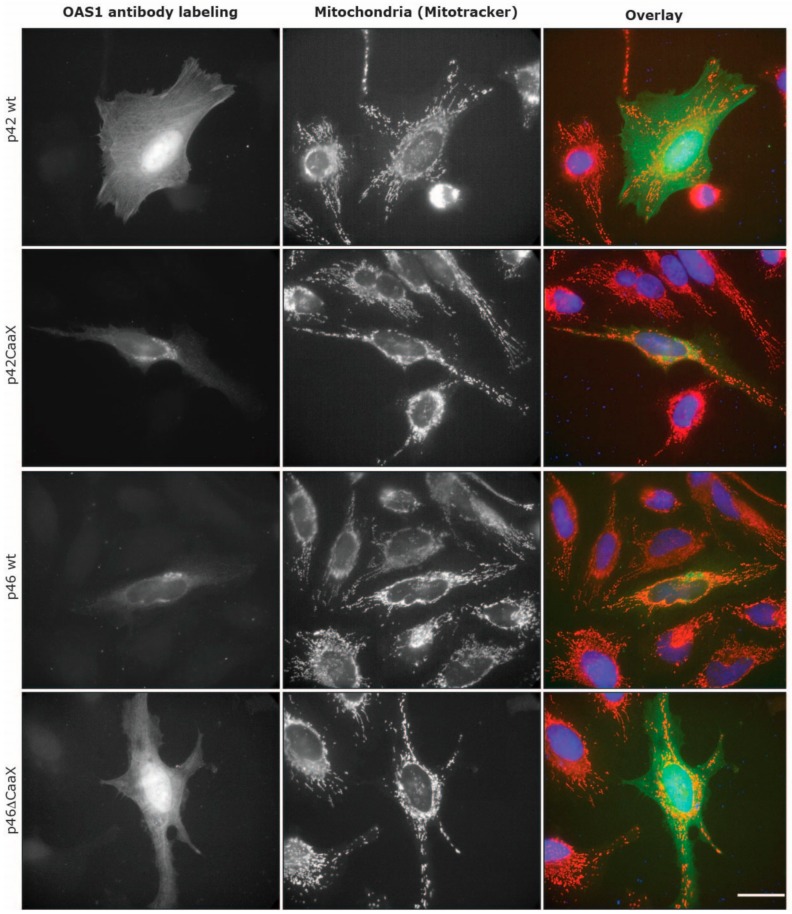
Cellular localization of OAS1 isoforms and mutants. HeLa cells were transfected with plasmids encoding the indicated OAS1 isoforms and mutants, p42 wt, p46 wt, p42CaaX, or p46ΔCaaX. Twenty-eight hours post transfection, cells were stained with Mitotracker (red) followed by immunocytochemistry analysis using the OAS1 antibody (green). The nuclei were stained with Hoechst 33342 (blue) and are shown in the overlay pictures. The scale bar is 30 µm.

Based on the difference in mitochondrial respiration upon overexpression of OAS1 p46 and p42 observed above, and the fact that we have previously observed the p46 isoform being associated with the mitochondria [9], we hypothesized that the localization of the OAS1 isoforms is of importance. We also hypothesized that a determining factor of subcellular localization was the CaaX motif only present on the C-terminus of the p46 isoform. To further elucidate the spatial distribution of OAS1 p46 and p42 and investigate the importance of CaaX, we constructed plasmids encoding p42 and p46 mutants, one in which the CaaX motif was deleted from p46 (p46ΔCaaX), and one in which the CaaX motif was added to the C-terminus of p42 (p42CaaX). A diagram of the different OAS1 constructs can be seen in Figure 1. Then we performed immunofluorescence microscopy using OAS1 antibodies on HeLa cells transfected with plasmids encoding p46 wt, p46ΔCaaX, p42 wt and p42CaaX (Figure 6). The overlaid images showed a clear correlation between having the CaaX motif and being situated in organelles in the perinuclear region. In this region, the OAS1 p46 wt with CaaX (green) is mainly co-localized with the red Mitotracker, while showing very little signal in the cytosolic space. However, when the CaaX motif is deleted in the p46ΔCaaX variant the spatial distribution completely changes into a more cytosolic associated protein. The exact opposite pattern was observed with the OAS1 p42 wt, being highly dispersed in the cytosol, but changing to a more organelle-associated protein when the CaaX motif was added. The same correlation between the CaaX motif and spatial distribution could be observed with endogenous OAS1 protein in IFN-β treated HeLa cells (only p42) and HT1080 cells (mainly p46) (Supplementary Figure S4).

**Figure 7:**
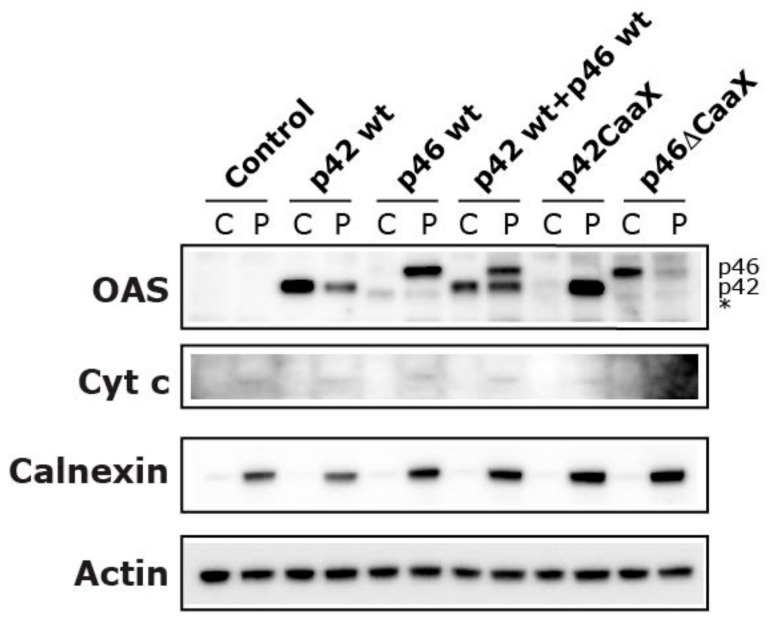
Cellular localization of OAS1 isoforms and mutants. HeLa cells were transfected with plasmids encoding one of the OAS1 isoforms and mutants, p42 wt, p46 wt, p42CaaX, p46ΔCAAX, p42, and p46 together (p42 wt + p46 wt), or a control plasmid. Twenty-four hours post transfection, cells were harvested by centrifugation. The cells were lysed and fractionated into the cytosol (C) and the pellet (P). The P fraction contains proteins from mitochondria, endoplasmic reticulum, Golgi and other internal organelles excluding the nucleus, which was separated in a centrifugation step. Protein extracts were subjected to immunoblot analysis using antibodies against the indicated proteins. The anti-OAS antibody recognizes all the OAS isoforms. The positions of the p42 and p46 isoforms are indicated. * Indicates the position of a cleaved form of p46.

To further substantiate these results, we performed an immunoblot analysis on subcellular fraction samples from transfected HeLa cells (Figure 7). In the fractionation protocol used, we separated the cytosolic (C) fraction from the pellet (P) fraction (containing membrane-enclosed organelles like the mitochondria, ER, and Golgi) by selectively permeabilizing the plasma membrane with digitonin. Figure 7 effectively illustrates that the OAS1 p46 isoform is almost entirely localized in the P fraction, whereas the majority of p42 resides in the cytosol. Again, either adding CaaX to p42 or deleting CaaX from p46 completely changes the localization of these isoforms, as the p42CaaX almost completely shifts to the P fraction while the p46 ΔCaaX shifts to the cytosolic fraction. Interestingly, the data from the simultaneously transfected p42+p46 sample showed that the distribution of p42 changed to being more localized in the P fraction compared to when p42 was expressed alone. This could mean that the p46 isoform might be able to facilitate the transport of some p42 isoform, perhaps in the tetrameric form.

### 3.4. Intracellular Localization of EGFP Fused to the C-Terminus of OAS1 p46 and p42

**Figure 8:**
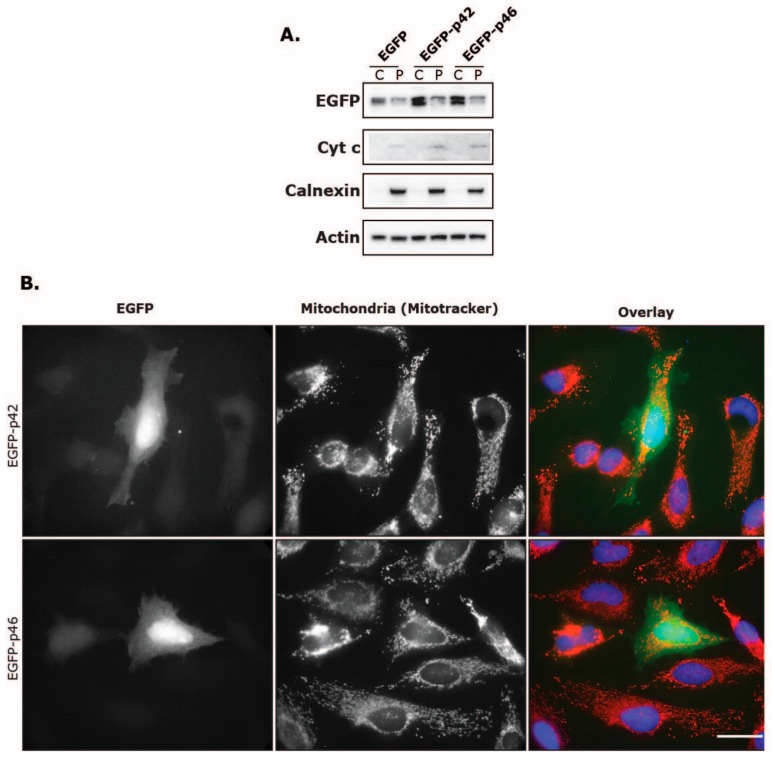
The C-terminal p46 CaaX motif cannot change the cellular localization of EGFP. HeLa cells were transfected with plamids encoding EGFP fused to either the p42 C-terminal end or the p46 C-terminal end, or a control EGFP expressing plasmid, as indicated. Twenty-eight hours after transfection, cells were (**A**) harvested by centrifugation for cell fractionation, or (**B**) stained with Mitotracker and subjected to immunocytochemistry analysis. (**A**) The cells were lysed and fractionated into the cytosol (C) and the pellet (P). The P fraction contains proteins from mitochondria, endoplasmic reticulum, Golgi and other internal organelles excluding the nucleus, which was separated in a centrifugation step. Protein extracts were subjected to immunoblot analysis using antibodies against the indicated proteins. (**B**) EGFP is green, the Mitotracker is red, and the nuclei were stained with Hoechst 33343 (blue) and are shown in the overlay pictures. The scale bar is 30 µm.

To further test the translocating capabilities of the CaaX motif, we fused either the p46 or the p42 C-terminus to an EGFP protein (Figure 1). This would determine if the CaaX motif in p46 by itself could change the cellular localization of EGFP. After transfection of HeLa cells with plasmids coding for either the EGFP-p42, EGFP-p46 or a control EGFP, we analyzed the fractionated samples by immunoblotting analysis (Figure 8A). In this experiment we observed that the EGFP was mainly localized in the cytosolic fraction, and we did not observe any apparent shift in distribution when the p42 or p46 C-terminus was fused to EGFP. We also analyzed this in HeLa cells using fluorescence microscopy (Figure 8B). Again, no difference in localization could be seen when comparing the EGFP-p42 and EGFP-p46. These results suggest that the C-terminus of OAS1 p46 and its CaaX motif is in itself not enough for membrane translocation, at least in this EGFP setup. 

## 4. Discussion

The interferon-induced OAS system is well described, at least regarding the molecular mechanisms involved, and the importance of the OAS1 proteins in the innate immune system and the protection against viral infections is evident. However, very little is known of the physiological impact of the OAS1 proteins on the mitochondria and the relationship between this and their subcellular localization. The localization and the pathophysiological mitochondrial effects of the major OAS1 isoforms p42 and p46 protein isoforms were therefore investigated in this study.

The respiration in HeLa and HT1080 cells was investigated using the high resolution respirometry (HRR) capabilities of the Oroboros Oxygraph-2k in different induced respiration states. Our results indicate that IFN-β treatment and OAS1 p42 overexpression in HeLa cells facilitate a significant increase in OCR at several respiration states. In HeLa cells, IFN-β (48 hours) increased the OCR in the ROUTINE, LEAK, and ETS states, as well as the ADP-stimulated state in the extended protocol, while OAS1 p42 overexpression only significantly increased the ETS state (compared to control and p46). IFN-β treatment also concomitantly increased the Δψm in HeLa cells in the CCCP titration steps. In HT1080 cells the 48 hours of IFN-β treatment only increased the ETS levels slightly, while OAS1 p42 overexpression increased this respiration state even more (when compared to control). Interestingly though, the overexpression of OAS1 p46 in these cells lowered the OCR significantly in the ROUTINE and ETS respiration states (compared to control and p42). Generally, IFN-β is known to promote glycolysis while impeding the OXPHOS resulting in the well-studied Warburg effect [29]. This phenomenon was observed after STAT1 expression in a human squamous carcinoma cell line and type I interferon treatment of murine L-929 cells, human Daudi lymphoblastoid cells and isolated CD4+ lymphocytes from MS patients [30,31,32]. However, an increase in OXPHOS has also been observed after type I interferon treatment in mouse plasmacytoid dendritic cells and non-hematopoietic cells [33], suggesting that the response is cell-type specific. This cell-type specificity might be influenced by the OAS1 A/G genotype, as the above-mentioned interferon treated Daudi cells (GG genotype) and our OAS1 p46 overexpressing HT1080 cells (AG genotype) both have decreased OCR, whereas the IFN-β treated and OAS1 p42 overexpressing HeLa cells (AA genotype) showed substantially increased OCR, while the IFN-β treated HT1080 cells only showed a slightly increased OCR. How this cell-type specificity results in a higher OCR in both HeLa and HT1080 cells when overexpressing p42, but only decreases by p46 overexpression in the HT1080 cells is interesting, as both cell lines seem to have the same p46 spatial distribution. In this context it is noticeable that in contrast to HeLa cells, the HT1080 cells showed a small OAS1 expression response after transfection with an empty vector as well as p46 expression in the cells transfected with the p42 expressing plasmid. The differences observed between the HeLa and HT1080 cells could also be influenced by the OAS2 and OAS3 distribution, which seems to be slightly different in the two cell lines, or by other Interferon Stimulated Genes (ISGs). It is noteworthy that these differences observed were without introducing foreign dsRNA, e.g., synthetic dsRNA like poly I:C or through a viral infection, which should elicit a more powerful response.

We have previously shown that the OAS1 p46 isoform is mainly found in the mitochondria whereas the p42 isoform is more widely distributed throughout the cytoplasm using electron microscopy [9]. We hypothesized that this translocation is based on the C-terminal CaaX motif only located on the OAS1 p46 isoform. In order to test this hypothesis mutant forms of p42 and p46 were created with and without the CaaX motif, and immunocytochemistry was performed with fluorescence microscopy to visualize the subcellular localizations of the different OAS1 variants. These results have also been substantiated by the cell fractionations and subsequent immunoblot analysis of both HT1080 and HeLa cells that have been treated with IFN-β or transiently transfected with the OAS1 expressing plasmids. In these experiments we clearly demonstrate that the OAS1 p42 isoform is mainly distributed evenly throughout the cytosol whereas the OAS1 p46 isoform is strongly associated with the membranous organelles (e.g., mitochondria and ER). Furthermore, we show for the first time that the CaaX motif located on the C-terminal on the p46 isoform, and its presumed prenylation, can fully facilitate this membrane translocation to the organelles. 

The amino acid sequence of the OAS1 p46 CaaX motif is CTIL and according to the literature, this would result in a geranylgeranyl type modification [34,35]. New evidence has recently emerged linking the geranylgeranyl modification with the transport of proteins into mitochondria-associated endoplasmic reticulum (ER) membranes (MAMs) at the mitochondria-ER junction upon viral infection [36]. Given the strong association of ER and mitochondria and the difficulty to discern between these structures in microscopy, and also the fact that our pellet fractions in the immunoblotting results also contains ER elements, we speculate that the MAMs at the mitochondria-ER junction may be a possible localization for the OAS1 p46 isoform. Several studies have previously observed a CaaX-mediated ER translocation of proteins [37,38,39].

The fact that the p46 CaaX motif was not enough in itself to facilitate the translocation of EGFP into the organelles could be explained by the observed tetramerization of the OAS1 protein [40], which is required for enzymatic activity. Perhaps this tetramerization in conjunction with the increase in geranylgeranyl lipids is important for the membrane association and could also explain the results showing that the OAS1 p46 isoform seemed to facilitate the translocation of the p42 isoform. In this case, it would then be a tetramer containing both isoforms. 

The HRR results presented here could support a model in which the cytosolic OAS1 p42 converts ATP into the 2′-5′ oligoadenylates and activates the RNase L. This can in turn activate RIG-I and MDA5 by the RNA cleavage products, and induce an interferon response through MAVS [41,42]. This pathway should indeed lead to an overall anti-viral resistance, but as stated previously the OAS1 p46 interestingly confers better protection against some forms of viral infection, which will be discussed shortly. IFN can, however, regulate many other pathways leading to the observed increase in respiration, e.g., increasing metabolic rates and substrates important for the electron donation to the ETS [29] or perhaps by increasing the amounts or capacity of the ETS complexes or components. The usage of ATP itself by OAS1 could also signal that OXPHOS should be increased, as ATP inhibits phosphofructokinase in the glycolysis. However, when OAS1 changes its localization, as happens with the p46 isoform, this also changes the effect on respiration. The exact molecular mechanisms driving this effect remain to be elucidated, however we hypothesize that there are two main components at play if OAS1 p46 can be transported into the mitochondrial matrix, which could be the case based on the HRR results in this study and the EM pictures in our previous study [9]. Firstly, the complete OAS1/RNase L system located in the mitochondria, would be able to degrade mitochondrial RNA encoding crucial proteins in the electron transport system, thereby indirectly inhibiting OXPHOS and mitochondrial function. Secondly, there might be a direct effect of synthesizing the 2′-5′ oligoadenylates, as this could consume and decrease the concentration of ATP in the inner matrix, thereby inhibiting the translocation of ADP through the ADP/ATP antiporter protein, adenine nucleotide translocase (ANT). This might create an imbalance in the ADP/ATP ratio, reduce the amount of ADP which is needed for ATP synthesis and thereby further decrease the levels of ATP concomitantly leading to the observed decrease in respiration and then perhaps subsequently resulting in apoptosis or necrosis [43].

This model would also serve to explain the observed differences in anti-viral protection between the OAS1 p46 and p42, as the p46 isoform would promote mitochondrial dysfunction and apoptosis, thereby inhibiting the viability and spread of viral pathogens. Furthermore, this could also explain the association of the SNP rs10774671 G allele, i.e., the OAS1 p46 variant, with multiple sclerosis (MS) and type 1 diabetes [44,45,46], as the mitochondrial dysfunction would drive both of these pathologies [47,48]. The OAS1 p46 isoform is then able to confer better anti-viral protection, while increasing the risk of mitochondrial dysfunction. However, there might be several mechanisms at play when elucidating the anti-viral difference between the OAS1 p46 and p42. 

If we entertain the idea that OAS1 p46 can be localized either in the ER membrane or in mitochondria closely connected with ER membranes, there could be several possible mechanisms conferring anti-viral protection. Viruses, and especially flaviviruses, usually associate with the ER for genome replication and the processing and assembly of viral proteins [49]. Therefore, this localization of OAS1 p46 could increase its interaction with these viruses. In fact, other than the binding of small dsRNAs, OAS1 can interact with the genome of WNV and hence be activated [50]. This interaction could be the key to the RNase L-independent anti-viral resistance previously observed using OAS1 mutants (in which the conservation of the RNA binding site was more important than the catalytic site in anti-viral activity) and the enzymatically inactive murine Oas1b, which was targeted to the ER and conferred WNV protection [51,52]. In addition, there is evidence that OAS1 can directly bind to the ER-associated NS5A protein from HCV [53] and that a CaaX motif can facilitate the interaction with NS5A [35], however in these cases the binding decreased the anti-viral effect, suggesting a defensive mechanism against the OAS1 protein. These observations could corroborate a mechanism in which the OAS1 p46 is located at the mitochondria-ER intersection and therefore in a higher proximity to viral RNA, which enables activation but also immobilization and inactivation of genomic and replicated viral RNA. This would confer better anti-viral protection and further explain the differences observed between the OAS1 p46 and p42 isoforms.

The proposed mechanisms are of course speculative at this point. The inherent differences between the OAS1 p46 and p42 isoforms in anti-viral activity and mitochondrial physiology are still largely unchartered territory and future studies must be pursued in order to get a more complete understanding. Nevertheless, we have in this study given novel insights into the spatial distribution and physiological effects of two of the major human OAS1 proteins, the p42 and p46 isoforms.

## Supplementary Materials

The following are available online at https://www.mdpi.com/1999-4915/11/12/1122/s1, Figure S1: High resolution respirometry (HRR) measurements of HeLa cells. Figure S2: Simultaneous OCR and fluorescence measurements in HeLa cells with or without IFN-β. Figure S3: Expression levels of p42 and p46 in HT1080 cells. Figure S4: Cellular localization of OAS1 in interferon treated HeLa cells and HT1080 cells.
