# Supplementary material for: The Cellular Localization of the p42 and p46 Oligoadenylate Synthetase 1 Isoforms and Their Impact on Mitochondrial Respiration
Source: Viruses. 2019 Dec 4;11(12):1122. doi: 10.3390/v11121122 (PMC6950736; doi:10.3390/v11121122)
Supplement: Supplementary file 1 [file viruses-11-01122-s001.pdf]

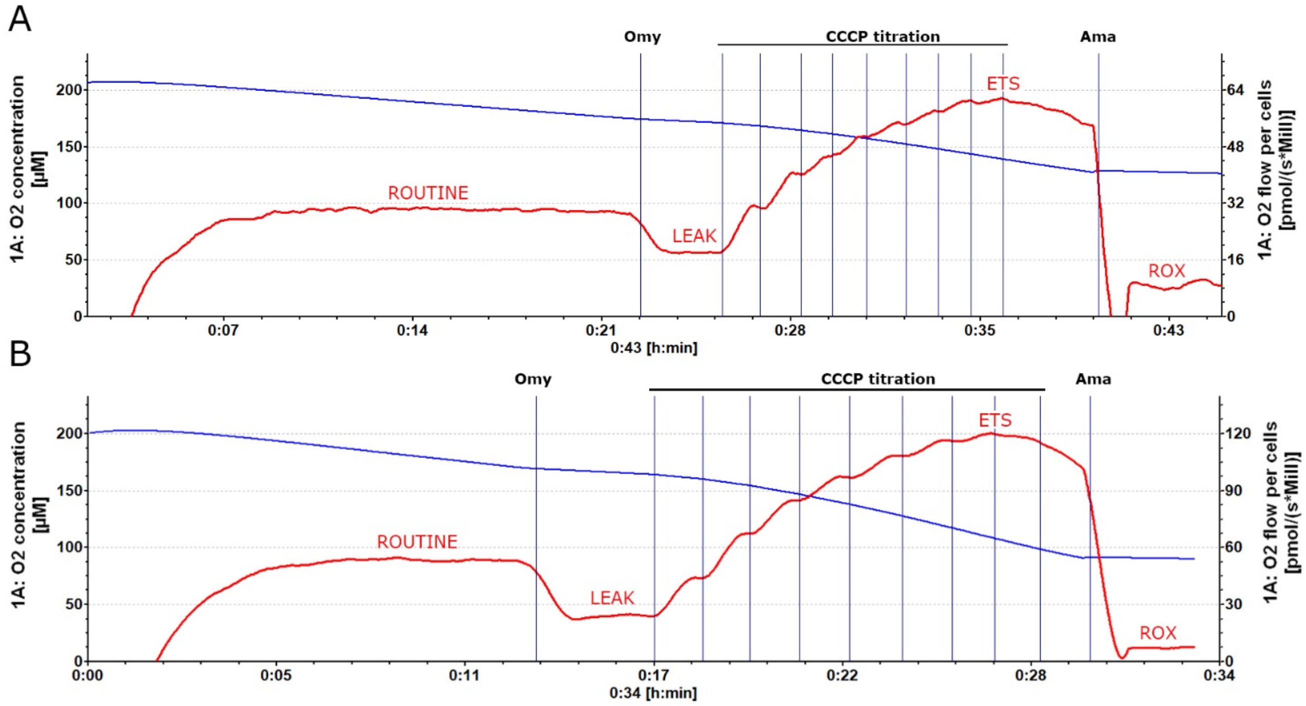

Figure S1: High resolution respirometry (HRR) measurements of HeLa cells. *Respiration of HeLa cells were measured after 48 hours without treatment (**A**) or with IFN-β treatment (**B**) using the substrate-uncoupler-inhibitor-titration (SUIT) protocol. ROUTINE, LEAK, ETS and ROX respiration states are indicated in red text. Black text and vertical lines indicate treatment with 2.5 µM oligomycin (Omy), 0.5 µM CCCP titration steps or 2.5 µM antimycin A (Ama). Red curves are oxygen consumption rate (OCR) in pmol O_2/sec/1 x 10^6 cells (right Y-axis) and blue curves are oxygen concentration in µM (left Y-axis). X-axis is time in hours and minutes (h:min). Note that OCR scales are substantially different between the two measurements.*

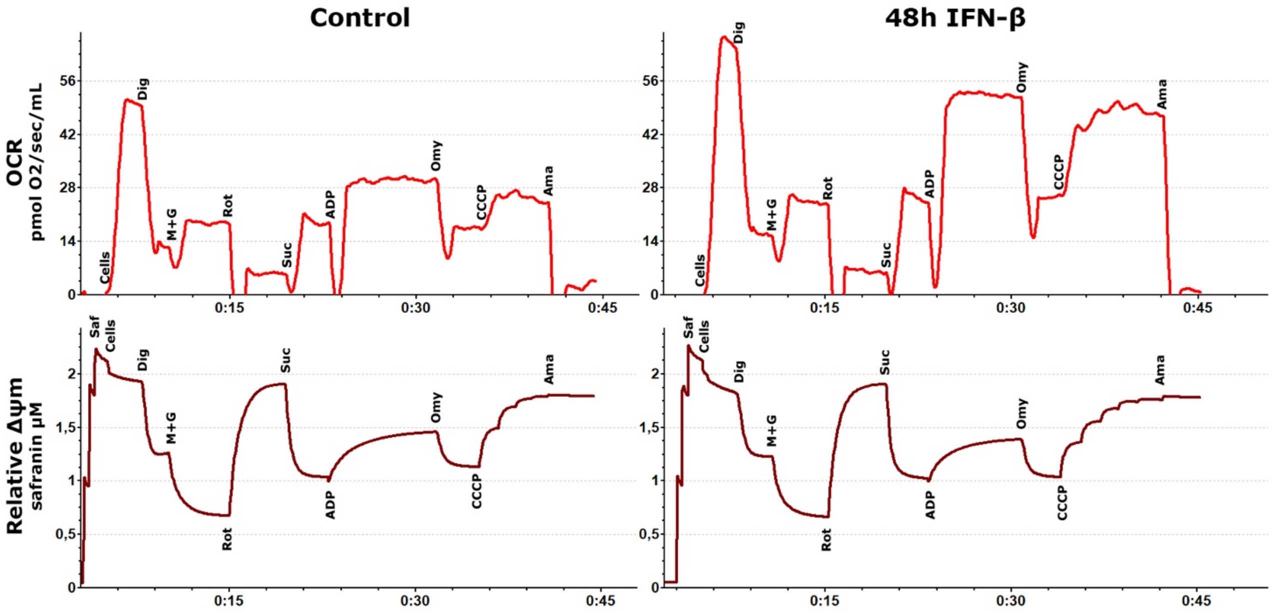

Figure S2: Simultaneous OCR and fluorescence measurements in HeLa cells with or without IFN-β.

The extended SUIT protocol is performed on HeLa cells either treated with IFN-β for 48 hours (right graphs) or left untreated (control) (left graphs). Injection of SUIT chemicals are indicated on all graphs. OCR values (red curves) are compared side by side (top graphs) with equal Y-axis scaling. The relative $\Delta\psi_m$ values (maroon curves) are compared side by side (bottom graphs) with equal Y-axis scaling. The X-axis represents timescale (h:min) which is equal for the OCR and relative $\Delta\psi_m$ in the respective treatments.

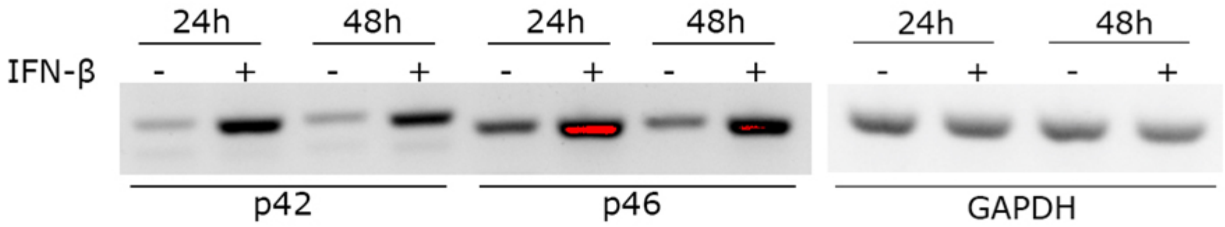

Figure S3: Expression levels of p42 and p46 in HT1080 cells. *RT-PCR of isolated RNA from HT1080 cells that were either treated with IFN-β (+) or left untreated (-) for 24h or 48h. The two isoforms were detected using specific primers for p42 and p46 in the PCR. GAPDH primers were used as a control. The red colour in the bands indicates saturated levels.*

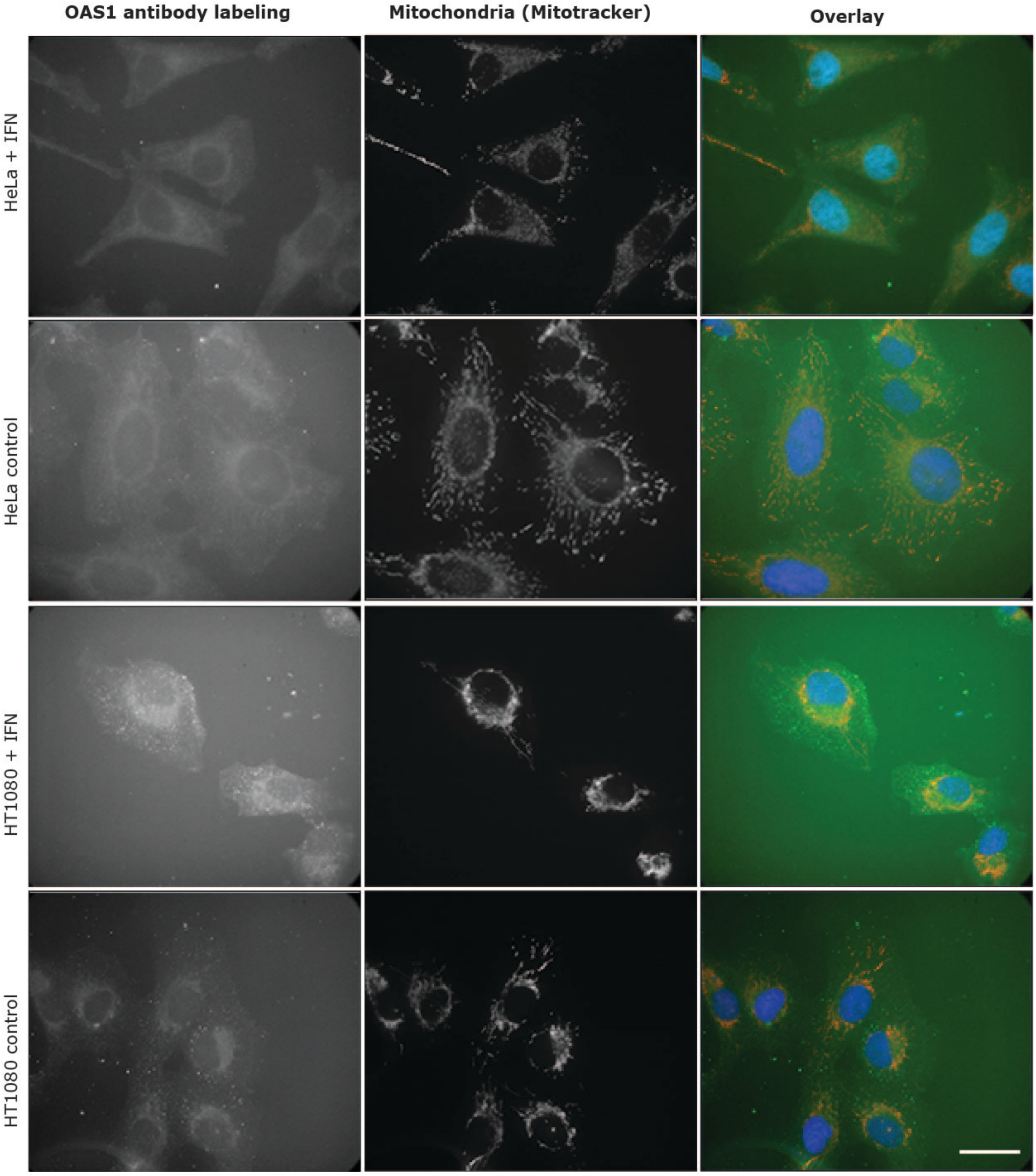

Figure S4: Cellular localization of OAS1 in interferon treated HeLa cells and HT1080 cells. *HeLa cells and HT1080 cells were stimulated with IFN-β for 28 hours. Cells were then stained with Mitotracker (red) followed by immunocytochemistry analysis using the OAS1 antibody (green). The nuclei were stained with Hoechst 33342 (blue) and are shown in the overlay pictures. The scale bar is 30 μm. Background for all images have been altered identically. All intensity below 800 have been removed. Differences in auto-fluorescent background is due to varying amounts of mounting media.*
